# Establishment and Validation of a Prognostic Risk Model for Autophagy-Related Genes in Clear Cell Renal Cell Carcinoma

**DOI:** 10.1155/2020/8841859

**Published:** 2020-11-10

**Authors:** Wenkai Han, Xiaoyan Xu, Kai Che, Guofeng Ma, Danxia Li, Mingxin Zhang, Wei Jiao, Haitao Niu

**Affiliations:** ^1^Department of Urology, The Affiliated Hospital of Qingdao University, Qingdao, Shandong, China; ^2^Department of Clinical Medicine, Qingdao University, Qingdao, Shandong, China

## Abstract

**Background:**

Autophagy plays an essential role in tumorigenesis. At present, due to the unclear role of autophagy in renal clear cell carcinoma, we studied the potential value of autophagy-related genes (ARGs) in renal clear cell carcinoma (ccRCC).

**Methods:**

We obtained all ccRCC data from The Cancer Genome Atlas (TCGA). We extracted the expression data of ARGs for difference analysis and carried out biological function analysis on the different results. The autophagy risk model was constructed. The 5-year survival rate was assessed using the model, and the predictive power of the model was evaluated from multiple perspectives. Cox regression analysis was use to assess whether the model could be an independent prognostic factor. Finally, the correlation between the model and clinical indicators is analyzed.

**Results:**

The patients were divided into the high-risk group and low-risk group according to the median of autophagy risk score, and the results showed that the prognosis of the low-risk group was better than that of a high-risk group. The validation results of external data sets show that our model has good predictive value for ccRCC patients. The model can be an independent prognostic factor. Finally, the results show that our model has a stable predictive ability.

**Conclusion:**

The autophagy gene model we constructed can be used as an excellent prognostic indicator for ccRCC. Our study provides the possibility of individualized and precise treatment for ccRCC patients.

## 1. Introduction

Clear cell renal cell carcinoma (ccRCC) is the most common type of cancer in renal cell carcinoma and one of the most common malignancies of the urinary system [1]. ccRCC has a high incidence, low therapeutic effect and poor prognosis [2]. The early detection rate of ccRCC patients is low due to inconspicuous early symptoms of ccRCC and the lack of accurate diagnostic markers. Therefore, finding new diagnostic indicators was urgently needed to improve the early diagnosis and treatment of ccRCC. Autophagy is a process whereby lysosomes are activated by various pathways to degrade intracellular substances. Under normal conditions, autophagy is a dynamic cycling process in which cell growth and self-survival maintain a balance [3]. Autophagy plays a dual role in cancer. Under normal conditions, autophagy maintains the body's balance, monitors the body's cells, and inhibits cell cancerization. In contrast to its antitumour role before tumour formation, autophagy is beneficial to tumour growth [4]. In recent years, autophagy is associated with a variety of cancers, and the autophagy gene has been used as a novel target for tumour therapy [5]. Studies have shown that autophagy plays a vital role in pancreatic, colorectal, and prostate cancers [2, 6, 7]. Studies have demonstrated that the activation of the autophagy pathway can promote the growth and development of ccRCC, and the results show that the pathway constructed by TRPM3 and mir-204 can promote the progress of ccRCC [8]. Autophagy can activate the epithelial-mesenchymal transition (EMT) to induce infiltration and metastasis of ccRCC, which reduces the clinical therapeutic effect. Therefore, autophagy can be utilized as a potential target for ccRCC treatment [9]. At present, although studies on autophagy in ccRCC have involved various aspects, studies on the overall autophagy gene are insufficient. Therefore, we are required to construct a model based on autophagy gene to improve the early detection and survival prognosis of ccRCC patients.

In this study, we analyzed the expression data of all ccRCC samples in the TCGA database and screened the differentially expressed autophagy-related genes (DEARGs). We conducted Gene Ontology (GO) biological enrichment analysis and Kyoto Encyclopedia of Genes and Genomes (KEGG) pathway analysis on the DEARGs to understand the biological functions of the DEARGs. DEARGs associated with survival were screened, and the risk models were so manufactured. According to the risk score of the model, the patients were divided into a high-risk group and low-risk group; the survival and risk curves were drawn to determine the accuracy of the model. A Cox regression analysis was used to determine whether the predictive model and clinical indicators could be used as independent prognostic factors, and the ROC curve was drawn to verify the accuracy. Finally, we assessed the correlation between the model and clinical indicators.

In summary, our model can reasonably predict the prognosis of ccRCC, and the risk score can be used as an independent prognostic factor. Moreover, the correlation between the model molecular tag and the clinic provides us with the possibility of further accurate and individualized treatment.

## 2. Material and Methods

### 2.1. Data Download

We downloaded the transcriptome and clinical data from all the ccRCC samples from the TCGA database (https://portal.gdc.cancer.gov/). There were 72 cases of normal renal tissue and 539 cases of ccRCC in the expression data. The transcriptome data are in the format of FPKM. Also, we downloaded all transcriptome data from the International Cancer Genome Consortium (ICGC) data for European patients. We got all the ARGs (232) from the Human Autophagy Database (HAD, http://www.autophagy.lu/); the expression information of all ARGs (222) in ccRCC data was extracted.

**Figure 1:**
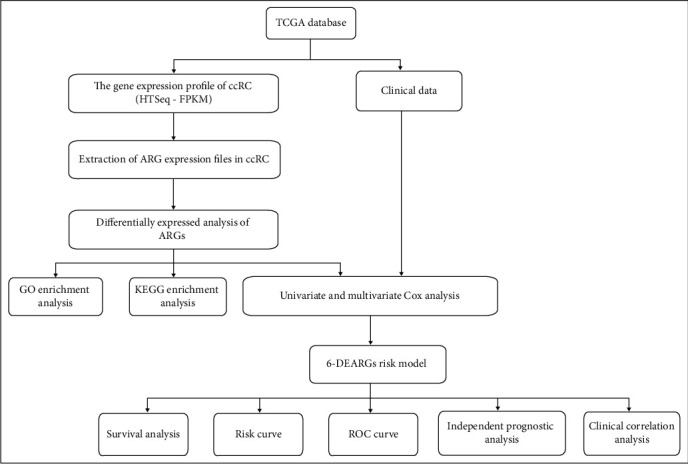
Flow chart of data analysis.

In this study, all of our analytical processes are shown in Figure 1.

### 2.2. DEARGs Were Selected from ccRCC

In order to screen DEARGs, we assessed the expression data of normal renal tissue and ccRCC. This operation is conducted in an R environment (version Rx64 3.6.2). The Limma package [10] was used to correct the data, and the Wilcoxon test was performed on the data (∣logFC | >1, FDR < 0.05). The result is visualized using the pheatmap package.

### 2.3. Biological Enrichment Analysis of DEARGs

In order to examine the biological function of DEARGs, we conducted GO and KEGG enrichment analysis on DEARGs. This operation runs in an R environment; the cutoff value was *p* < 0.5 and adjusted *p* < 0.05. The R package used for this operation includes “clusterProfiler” [11], “org.Hs.eg.db,” “enrichplot,” “DOSE,” “ggplot2,” “stringi,” “colorspace,” “digest,” and “GOplot.”

### 2.4. Construction Prognostic Risk Model

First, we screened DEARGs caused by the ccRCC prognosis. In the R environment, the “survival” package performed a univariate Cox regression analysis on DEARGs. We screened 8 DEARGs connected with the ccRCC prognosis. Then, we performed a multivariate Cox regression analysis on 8-DEARGs, and the results obtained a 5-DEARGs risk prediction model (*p* < 0.05). Depending on the model, we obtained a comprehensive prognostic scoring system (risk score). Riskscore = EXP_RNA1_ × *β*_RNA1_ + EXP_RNA2_ × *β*_RNA2_ + ⋯+EXP_RNAn_ × *β*_RNAn_, where EXP is the DEARGs expression level and *β* is the multivariate regression coefficient of the Cox regression model.

### 2.5. Survival Curve and Risk Curve

The patients with ccRCC were subdivided into a high-risk group and low-risk group according to the median risk score of the model. In the R environment, the “survival” and “survminer” packages were used for survival analysis and data visualization of patients in the high-risk and low-risk groups. We visualized the risk model, the survival state of ccRCC, and the risk curve according to the risk score of the model.

### 2.6. Validation of the Model

To further verify the accuracy of our model predictions, we validated the EU group of patients in the ICGC database. We divided patients into the high-risk and low-risk groups based on the median value of the model risk score. The survival status of the high-risk and low-risk groups was further observed. Besides, we draw ROC curves to assess the accuracy of model predictions. In order to further verify the expression of model genes in ccRCC, we used the Human Protein Atlas database (http://www.proteinatlas.org/) for verification.

### 2.7. Clinical Relevance of Risk Models

To further investigate the model, we discussed the correlation between the model and clinical traits. In the R environment, the “beeswarm” package was used for the *t*-test analysis of the data (*p* < 0.05).

### 2.8. Independent Prognostic Analysis and Validation

To further validate the feasibility of the risk model, we determined whether the risk score could be employed as an independent prognostic factor. We combined the clinical traits (survival time, survival status, age, gender, grade, stage, TNM stage) with the risk scores for the univariate (*p* < 0.05) and multivariate (*p* < 0.05) Cox analyses. We were visualizing data using the “survival” package in the R environment. Finally, through the “survivvalroc” package, we plotted the ROC curve of risk score and clinical traits to judge the accuracy of each indicator.

## 3. Results

### 3.1. Differential Expression Analysis of ARGs

**Figure 2:**
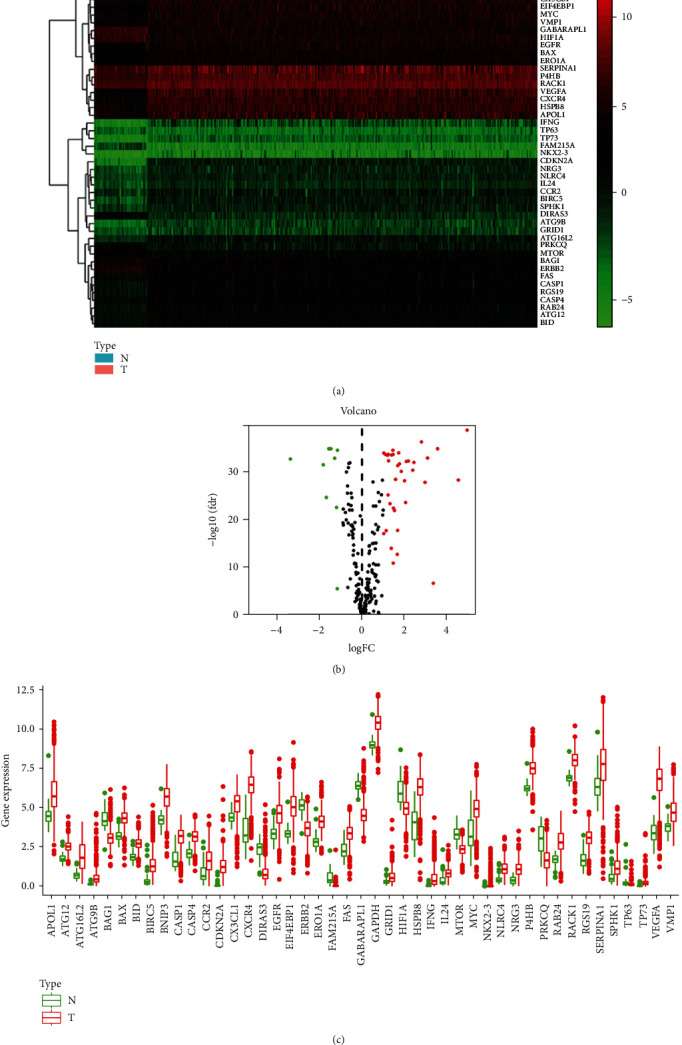
Differential expression of autophagy genes in ccRCC samples. (a) Heat map of 45 differential autophagy-related genes in the tumour and normal tissue samples. (b) Volcano plot of 45 differentially expressed autophagy-related genes. Red represents the high expression of autophagy-related genes, and green represents the low expression of autophagy-related genes. (c) Differential expression of autophagy-related genes in the tumour and normal tissue samples. Heat map of 45 differential autophagy genes.

We screened the expression information of 222 ARGs in ccRCC according to the 232 ARGs in HAD (Supplementary Table 1). According to the screening criteria (∣logFC | >1, FDR < 0.05), we finally got 45 DEARGs, including 9 downregulated and 36 upregulated ARGs. We visualized the data in the form of a heat map, volcano plot, and boxplot (Figure 2). The logFC in ccRCC is illustrated in Supplementary Table 2.

### 3.2. Enrichment Analysis of DEARGs in ccRCC

**Figure 3:**
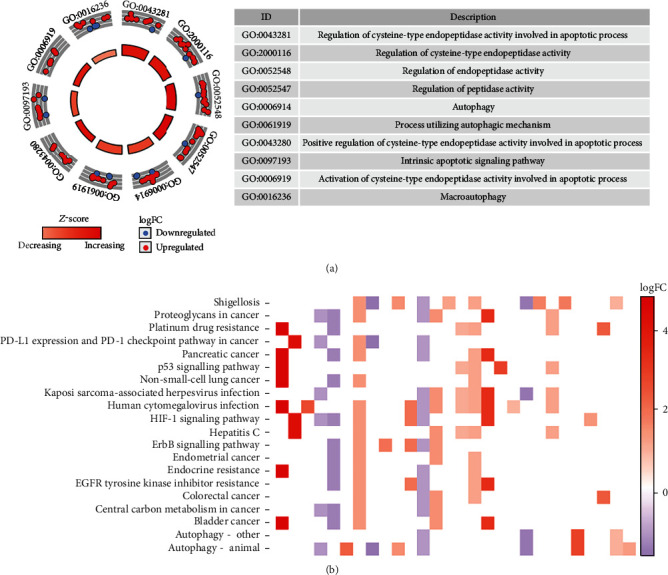
Biological function analysis of differentially expressed autophagy genes. (a) GO enrichment analysis of differential autophagy-related genes. (b) KEGG enrichment analysis of different autophagy-related genes.

To further study the biological function of DEARGs in ccRCC, we analyzed 45 DEARGs by GO and KEGG.  Figure 3(a) indicates the top 10 terms of the GO enrichment analysis results. The results showed that DEARGs were related to the regulation of cysteine-type endopeptidase activity involved in apoptotic process, regulation of cysteine-type endopeptidase activity, regulation of endopeptidase activity, regulation of peptidase activity, autophagy, the process utilizing autophagic mechanism, positive regulation of cysteine-type endopeptidase activity involved in the apoptotic process, intrinsic apoptotic signalling pathway, activation of cysteine-type endopeptidase activity involved in the apoptotic process, and macroautophagy.  Figure 3(b) shows that DEARGs in KEGG are related to autophagy–animal, autophagy–other, bladder cancer, central carbon metabolism in cancer, colorectal cancer, EGFR tyrosine kinase inhibitor resistance, endocrine resistance, endometrial cancer, ErbB signalling pathway, hepatitis C, HIF–1 signaling pathway, human cytomegalovirus infection, Kaposi sarcoma-associated herpesvirus infection, non-small-cell lung cancer, p53 signalling pathway, pancreatic cancer, PD–L1 expression and PD–1 checkpoint pathway in cancer, platinum drug resistance, proteoglycans in cancer, and Shigellosis pathway.

### 3.3. Identification of Prognostic DEARGs

In order to construct a prognostic survival risk model, we conducted a univariate Cox regression analysis of DEARGs, which selected 8-DEARGs associated with prognostic survival. The results are detailed in Supplementary Table 3. Among the genes we screened for 8-DEARGs, there were two low-risk genes and six high-risk genes.

### 3.4. Construction of Prognostic Risk Models

**Figure 4:**
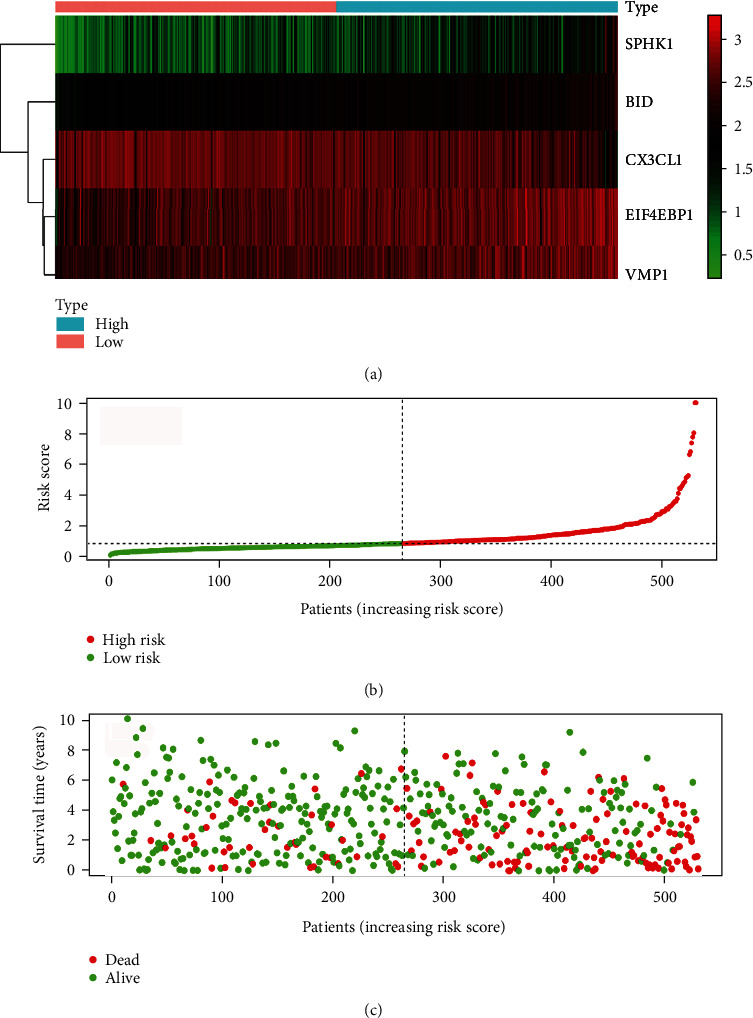
The prognostic signature in ccRCC. (a) The panel is a heat map of 5 genes. (b) The panel is the survival status and overall survival time of each ccRCC. (c) The panel is the risk score for each ccRCC.

In order to construct a prognostic risk model associated with autophagy genes, we conducted a multivariate Cox regression analysis of 8 genes associated with prognosis. Finally, we have a 5-gene prognostic risk model (Figure 4). The risk scores were calculated using correlations for 5-DEARGs (BID, CX3CL1, EIF4EBP1, VMP1, SPHK1). Riskscore = 0.6544 × EXP_BID_ − 0.2684 × EXP_CX3CL1_ + 0.1526 × EXP_EIF4EBP1_ + 0.2780 × EXP_VMP1_ + 0.1581 × EXP_SPHK1_. The risk score of patients is shown in Supplementary Table 4.

### 3.5. Survival Analysis of Autophagy Scores

**Figure 5:**
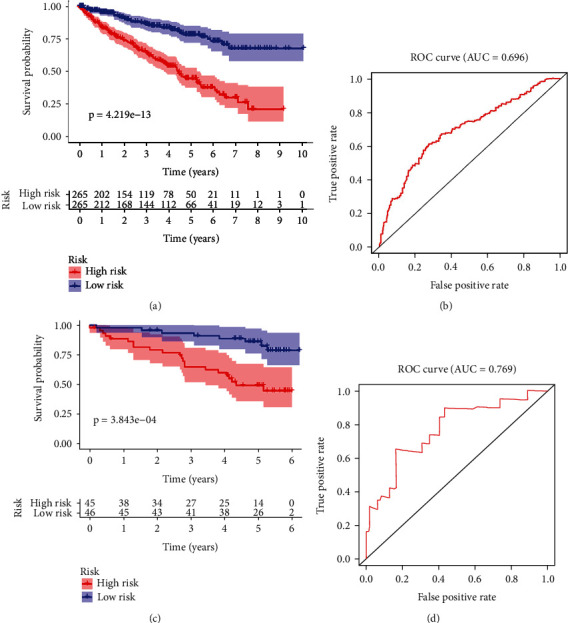
The prognostic signature in ccRCC. (a) The panel represents the overall survival of the sample at high and low risk. (b) The panel represents the ROC analysis. (c) The 5-year survival in the high-risk and low-risk groups in the EU group. (d) The panel represents the ROC analysis of the EU group.

We calculated the autophagy risk score for each patient based on the model. We divided patients into high-risk and low-risk groups based on the median autophagy risk score. Survival analysis was performed for both groups, and the results are shown in Figure 5(a). Then, we draw a ROC curve to assess the accuracy of the risk model (Figure 5(b)). The 5-year survival of the low-risk group was better than that of the high-risk group.

### 3.6. Validation of the Model

**Figure 6:**
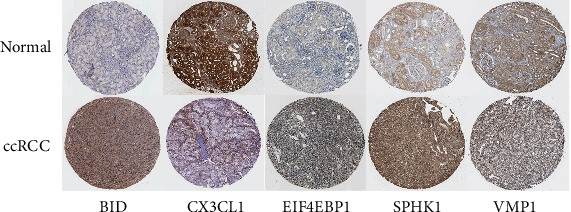
Immunohistochemical results of the ccRCC tissue and normal renal tissue. The expression of BID, CX3CL1, EIF4EBP1, VMP1, and SPHK1 in tumor and normal tissues.

Our model was validated using the EU group of patients in the ICGC database. According to the median risk score of our model, patients were divided into the high-risk and low-risk groups. The results showed that the patients in the high-risk group had a worse prognosis than those in the low-risk group (Figure 5(c)). Also, the ROC curve shows that our model has an excellent predictive effect (Figure 5(d)). We validated our model using the Human Protein Atlas database. The results showed that BID, EIF4EBP1, and SPHK1 were significantly increased in the ccRCC compared with the normal renal tissue. However, the staining level of CX3CL1 in the ccRCC tissues was relatively lower. There was no significant difference in VMP1 between the normal and tumour tissues. The results of immunohistochemistry were consistent with those of our analysis, which further confirmed the accuracy of our model (Figure 6).

### 3.7. Correlation Analysis between the Risk Models and Clinical Indicators

We further analyzed the correlation between the clinical with the gene signature in the model. BID was significantly associated with ccRCC grade (*p* = 7.631e − 05), stage (*p* = 7.868e − 08), T stage (*p* = 1.39e − 05), M stage (*p* = 0.006), and N stage (*p* = 7.43e − 05). CX3CL1 was significantly correlated with gender (*p* = 0.002), ccRCC grade (*p* = 4.402e − 04), stage (*p* = 3.66e − 04), and T stage (*p* = 1.761e − 04). EIF4EBP1 was significantly correlated with ccRCC age (*p* = 0.027), grade (*p* = 0.001), stage (*p* = 1.519e − 05), T stage (*p* = 2.761e − 04), M stage (*p* = 3.433e − 04), and N stage (*p* = 0.017). VMP1 was significantly associated with gender (*p* = 0.026), stage (*p* = 0.038), and T stage (*p* = 0.017). SPHK1 was significantly associated with ccRCC grade (*p* = 1.713e − 06), stage (*p* = 2.587e − 05), T stage (*p* = 1.237e − 04), M stage (*p* = 0.001), and N stage (*p* = 0.006). Risk score was significantly correlated with ccRCC grade (*p* = 1.874e − 06), stage (*p* = 2.489e − 06), T stage (*p* = 1.751e − 06), M stage (*p* = 0.019), N stage (*p* = 0.019), and M stage (*p* = 0.011) (supplementary Table 5).

### 3.8. Independent Prognostic Analysis

**Figure 7:**
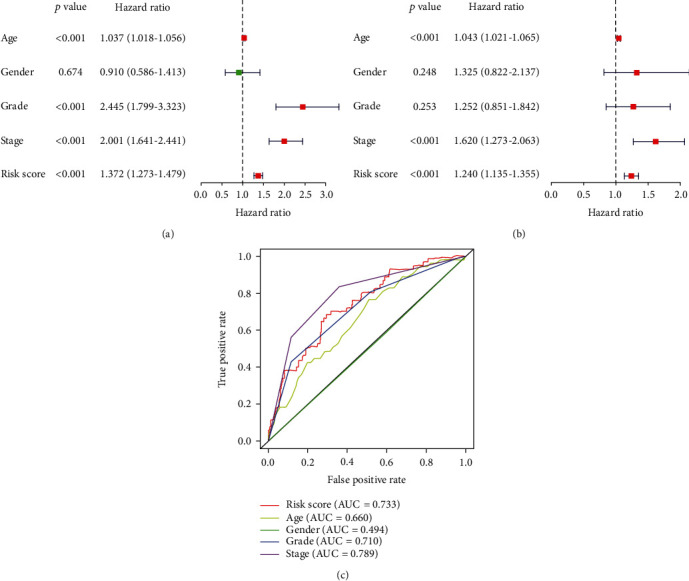
The model was combined with a regression analysis of clinical indicators: (a) univariate regression analysis; (b) multivariate regression analysis; (c) multi-index ROC curve.

**Figure 8:**
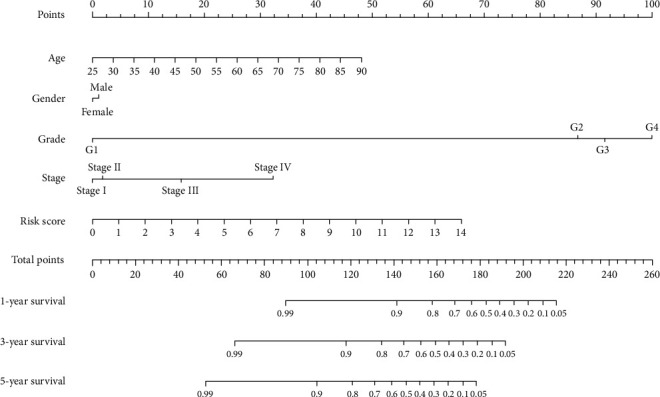
Clinicopathologic features of the ccRCC and prognostic nomograms of risk models.

We combined the autophagy risk score with clinical characteristics for Cox regression analysis, and the results showed that our autophagy risk score could be an independent prognostic factor for ccRCC (Figures 7(a) and 7(b)). We found that tumour grade can also be used as an independent predictor of postanalysis. Multi-indicator ROC curve shows that the autophagy risk score can effectively predict the prognosis of patients (Figure 8(c)). Also, our autophagy risk score, combined with clinical indicators, can effectively predict the survival status of ccRCC (Figure 8).

## 4. Discussion

Clear cell renal cell carcinoma (ccRCC) is the most common type of renal cell carcinoma and one of the most common tumours in the urinary system. In ccRCC patients, the early symptoms are not visible; most of them are detected by physical examination, and the lack of specific diagnostic markers makes early diagnosis difficult. Therefore, new diagnostic indicators are critical to the early detection and treatment of ccRCC.

Autophagy is critical at maintaining a stable intracellular environment [12]. Early studies have reported that autophagy has a significant relationship with the occurrence and progression of tumours [13, 14]. Autophagy also plays a major role in the occurrence and development of ccRCC [15]. Therefore, we investigated the role of autophagy genes in ccRCC and screened out autophagy genes that can be used as diagnostic indicators to improve the early diagnosis and prognostic survival of ccRCC.

In this study, RNA-seq data in the TCGA database were analyzed to screen ARGs related to ccRCC prognosis. We finally got 45 DEARGs, including 9 downregulated and 36 upregulated ARGs. In order to explore the biological function of DEARGs, we conducted GO and KEGG enrichment analysis on DEARGs. The results showed that these differentially expressed genes were closely related to autophagy, apoptosis, and bioregulation. These functions are closely associated with the occurrence and development of tumours. The KEGG enrichment analysis reveals that DEARGS is mainly related to the PD–L1 expression and PD–1 checkpoint pathway in cancer, p53 signalling pathway, HIF–1 signalling pathway, ErbB signalling pathway, EGFR tyrosine kinase inhibitor resistance, and autophagy pathway. The programmed cell death 1 receptor (PD–1) can be used as an immunoassay site for ccRCC to influence the ccRCC survival rate [16]. Besides, numerous other pathways have been reported in ccRCC [17–19]. Univariate Cox analysis was performed on DEARGs to screen for autophagy genes associated with survival. We screened 8 autophagy genes associated with survival (BIRC5, BID, SPHK1, CX3CL1, EIF4EBP1, VMP1, BAG1, CASP4). Then, we performed a multivariate Cox regression analysis on 8-DEARGs, and obtained a 5-DEARGs (BID, CX3CL1, EIF4EBP1, VMP1, SPHK1) risk prediction model (*p* < 0.05). According to the autophagy risk score, the patients were subdivided into the high-risk and low-risk groups. The survival analysis showed that the 5-year survival rate was significantly lower in the high-risk group than in the low-risk group. The ROC analysis also reflects the accuracy of an autophagy risk model. The correlation analysis between the model with clinical traits showed that the tumour markers were associated with age, gender, grade, stage, and TMN stage. The correlation analysis also verified the reliability of our autophagy risk signature. Finally, we analyzed whether the autophagy risk score and clinical traits could be independent prognostic factors. The results showed that the autophagy risk model could be an independent factor for ccRCC prognostic survival, and the results of ROC analysis also demonstrated the feasibility of the model as an independent prognosis (riskscore_AUC_ = 0.733, grade_AUC_ = 0.710, and stage_AUC_ = 0.789).

In summary, our study indicates that autophagy gene plays an essential role in ccRCC. The autophagy gene-related risk prediction model developed by us has good accuracy for ccRCC. Our study provides the possibility of more precise research and clinical treatment. In our risk model, BID, EIF4EBP1, VMP1, and SPHK1 were high-risk genes, while CX3CL1 was low-risk genes. BID promotes the occurrence and metastasis of ccRCC by altering TNF signals [20]. Other studies have shown that BID is closely related to the survival prognosis of ccRCC [21–23]. SPHK1 can be used as a target gene of HIF-1 to influence the prognosis of ccRCC [24]. The SphK1 overexpression promotes the RCC process by regulating the Akt/mTOR pathway [25]. CX3CL1 is closely related to the metastasis and invasion of ccRCC [26]. It has been reported that BID and EIF4EBP1 can be used as predictive model markers of ccRCC [22]. Unfortunately, there has been no study of VMP1 in ccRCC. Although no studies have been conducted on VMP1 and ccRCC, VMP1, as an autophagy gene, plays a vital role in the development and invasion of pancreatic cancer and liver cancer [27, 28]. Autophagy-related genes BID, EIF4EBP1, VMP1, SPHK1, and CX3CL1 also play essential roles in other cancers.

## 5. Conclusion

In this study, we used the autophagy gene expression data of ccRCC in TCGA data to build a risk prediction model, and we verified the accuracy of our model through several aspects. However, we need to acknowledge the limitations of this study, and the following points need to be explained: (1) Our model needs to be validated in a prospective clinical trial. (2) At present, the research on autophagy-related genes is not mature and still needs further research to improve. We firmly believe that the diagnosis and treatment of ccRCC are becoming more and more perfect with the deepening of research.

## Data Availability

We downloaded transcriptome and clinical data from all ccRCC samples from the TCGA database (https://portal.gdc.cancer.gov/). There were 72 cases of normal renal tissue and 539 cases of ccRCC in the expression data. The transcriptome data are in the format of FPKM. Also, we downloaded all transcriptome data from the International Cancer Genome Consortium (ICGC) data for European patients. We got all the ARGs (232) from the Human Autophagy Database (HADb, http://www.autophagy.lu/); the expression information of all ARGs (222) in ccRCC data was extracted.

## Supplementary Materials

Supplementary 1 Supplementary table S1 There are 232 autophagy related genes in HAD.

Supplementary 2 Supplementary Table S2 LogFC expression levels of different autophagy related genes in ccRCC.

Supplementary 3 Supplementary Table S3 Univariate Cox regression analysis of DEARGs.

Supplementary 4 Supplementary Table S4 Multivariate Cox regression analysis of 8 genes.

Supplementary 5 Supplementary Table S5 The correlation between gene signature and clinical traits.
