# Supplementary material for: Establishment and Validation of a Prognostic Risk Model for Autophagy-Related Genes in Clear Cell Renal Cell Carcinoma
Source: Dis Markers. 2020 Nov 10;2020:8841859. doi: 10.1155/2020/8841859 (PMC7676277; doi:10.1155/2020/8841859)
Supplement: Supplementary 1 — Supplementary table S1 There are 232 autophagy related genes in HAD. [file 8841859.f1.docx]

| Name | Gene Symbol |
| --- | --- |
| ARGs | AMBRA1 APOL1 ARNT ARSA ARSB ATF4 ATF6 ATG10 ATG12 ATG16L1 ATG16L2 ATG2A ATG2B ATG3 ATG4A ATG4B ATG4C ATG4D ATG5 ATG7 ATG9A ATG9B ATIC BAG1 BAG3 BAK1 BAX BCL2 BCL2L1 BECN1 BID BIRC5 BIRC6 BNIP1 BNIP3 BNIP3L C12orf44 C17orf88 CALCOCO2 CAMKK2 CANX CAPN1 CAPN10 CAPN2 CAPNS1 CASP1 CASP3 CASP4 CASP8 CCL2 CCR2 CD46 CDKN1A CDKN1B CDKN2A CFLAR CHMP2B CHMP4B CLN3 CTSB CTSD CTSL1 CX3CL1 CXCR4 DAPK1 DAPK2 DDIT3 DIRAS3 DLC1 DNAJB1 DNAJB9 DRAM1 EDEM1 EEF2 EEF2K EGFR EIF2AK2 EIF2AK3 EIF2S1 EIF4EBP1 EIF4G1 ERBB2 ERN1 ERO1L FADD FAM48A FAS FKBP1A FKBP1B FOS FOXO1 FOXO3 GAA GAA GABARAP GABARAP GABARAPL1 GABARAPL1 GABARAPL2 GABARAPL2 GAPDH GAPDH GNAI3 GNAI3 GNB2L1 GNB2L1 GOPC GOPC GRID1 GRID1 GRID2 GRID2 HDAC1 HDAC6 HGS HIF1A HSP90AB1 HSPA5 HSPA8 HSPB8 IFNG IKBKB IKBKE IL24 IRGM ITGA3 ITGA6 ITGB1 ITGB4 ITPR1 KIAA0226 KIAA0652 KIAA0831 KIF5B KLHL24 LAMP1 LAMP2 MAP1LC3A MAP1LC3B MAP1LC3C MAP2K7 MAPK1 MAPK3 MAPK8 MAPK8IP1 MAPK9 MBTPS2 MLST8 MTMR14 MTOR MYC NAF1 NAMPT NBR1 NCKAP1 NFE2L2 NFKB1 NKX2-3 NLRC4 NPC1 NRG1 NRG2 NRG3 P4HB PARK2 PARP1 PEA15 PELP1 PEX14 PEX3 PIK3C3 PIK3R4 PINK1 PPP1R15A PRKAB1 PRKAR1A PRKCD PRKCQ PTEN PTK6 RAB11A RAB1A RAB24 RAB33B RAB5A RAB7A RAC1 RAF1 RB1 RB1CC1 RELA RGS19 RHEB RPS6KB1 RPTOR SAR1A SERPINA1 SESN2 SH3GLB1 SIRT1 SIRT2 SPHK1 SPNS1 SQSTM1 ST13 STK11 TBK1 TM9SF1 TMEM49 TMEM74 TNFSF10 TP53 TP53INP2 TP63 TP73 TSC1 TSC2 TUSC1 ULK1 ULK2 ULK3 USP10 UVRAG VAMP3 VAMP7 VEGFA WDFY3 WDR45 WDR45L WIPI1 WIPI2 ZFYVE1 |

ARGs (232) from the Human Autophagy Database
