# Supplementary material for: Establishment and Validation of a Prognostic Risk Model for Autophagy-Related Genes in Clear Cell Renal Cell Carcinoma
Source: Dis Markers. 2020 Nov 10;2020:8841859. doi: 10.1155/2020/8841859 (PMC7676277; doi:10.1155/2020/8841859)
Supplement: Supplementary 2 — Supplementary Table S2 LogFC expression levels of different autophagy related genes in ccRCC. [file 8841859.f2.docx]

**Results of difference analysis**

| Gene | ConMean | TreatMean | LogFC | PValue | Fdr |
| --- | --- | --- | --- | --- | --- |
| ATG9B | 0.11239 | 0.869842 | 2.952246 | 2.52E-29 | 1.51E-28 |
| ATG16L2 | 0.670101 | 3.480231 | 2.376732 | 5.17E-32 | 3.97E-31 |
| RGS19 | 2.453338 | 8.046626 | 1.713638 | 3.22E-36 | 8.64E-35 |
| HSPB8 | 19.2273 | 86.7659 | 2.173972 | 4.14E-34 | 4.69E-33 |
| VEGFA | 10.79032 | 125.7155 | 3.542354 | 2.71E-37 | 1.29E-35 |
| BAX | 8.618243 | 20.25856 | 1.233066 | 9.01E-36 | 1.94E-34 |
| NLRC4 | 0.380052 | 1.354478 | 1.833468 | 1.00E-31 | 7.45E-31 |
| SERPINA1 | 106.3145 | 333.4466 | 1.649118 | 7.83E-14 | 1.75E-13 |
| ERO1A | 6.665222 | 18.30562 | 1.457562 | 1.04E-35 | 1.96E-34 |
| CCR2 | 1.05001 | 2.717555 | 1.371906 | 4.03E-15 | 9.62E-15 |
| GABARAPL1 | 84.36011 | 28.69902 | -1.55556 | 3.00E-37 | 1.29E-35 |
| CXCR4 | 13.70919 | 94.82624 | 2.790143 | 4.33E-39 | 4.65E-37 |
| TP63 | 0.258764 | 0.115188 | -1.16765 | 1.94E-06 | 3.07E-06 |
| CASP1 | 2.518214 | 8.464013 | 1.748941 | 2.10E-33 | 1.88E-32 |
| MYC | 12.39718 | 35.53526 | 1.51924 | 3.09E-23 | 1.15E-22 |
| FAM215A | 0.54183 | 0.052769 | -3.36008 | 1.44E-34 | 1.83E-33 |
| BAG1 | 20.13346 | 8.229862 | -1.29066 | 9.52E-35 | 1.28E-33 |
| BNIP3 | 18.27418 | 54.2752 | 1.570487 | 5.11E-30 | 3.54E-29 |
| BIRC5 | 0.401041 | 2.170156 | 2.435978 | 9.38E-34 | 9.60E-33 |
| TP73 | 0.02929 | 0.245782 | 3.068905 | 7.64E-35 | 1.10E-33 |
| ATG12 | 2.466337 | 5.002604 | 1.020309 | 4.17E-36 | 9.97E-35 |
| BID | 2.750228 | 5.892019 | 1.099211 | 1.10E-35 | 1.96E-34 |
| GAPDH | 522.8686 | 1428.527 | 1.450008 | 7.25E-37 | 2.55E-35 |
| ERBB2 | 35.63789 | 12.91565 | -1.46429 | 2.79E-37 | 1.29E-35 |
| PRKCQ | 8.453836 | 2.651548 | -1.67277 | 4.39E-26 | 2.19E-25 |
| CX3CL1 | 20.3831 | 41.1572 | 1.013771 | 3.02E-18 | 8.33E-18 |
| DIRAS3 | 4.383877 | 1.240959 | -1.82075 | 3.48E-33 | 2.99E-32 |
| EIF4EBP1 | 9.736555 | 41.00997 | 2.074491 | 5.29E-34 | 5.69E-33 |
| CDKN2A | 0.063563 | 1.927831 | 4.922641 | 7.21E-42 | 1.55E-39 |
| IFNG | 0.033549 | 0.77013 | 4.520773 | 6.90E-30 | 4.64E-29 |
| P4HB | 77.15507 | 201.3147 | 1.38362 | 1.51E-35 | 2.50E-34 |
| RAB24 | 2.272892 | 7.245195 | 1.672495 | 5.39E-33 | 4.46E-32 |
| GRID1 | 0.243958 | 0.673373 | 1.464774 | 5.80E-12 | 1.27E-11 |
| EGFR | 10.2383 | 28.39875 | 1.471851 | 8.44E-24 | 3.42E-23 |
| MTOR | 9.448338 | 4.229551 | -1.15956 | 8.30E-37 | 2.55E-35 |
| NKX2-3 | 0.013388 | 0.135627 | 3.340627 | 1.35E-07 | 2.30E-07 |
| HIF1A | 77.61493 | 33.72897 | -1.20235 | 6.60E-24 | 2.73E-23 |
| CASP4 | 3.514194 | 8.346382 | 1.247957 | 3.45E-34 | 4.12E-33 |
| APOL1 | 25.99648 | 107.0531 | 2.041938 | 5.05E-25 | 2.36E-24 |
| VMP1 | 13.10452 | 32.49707 | 1.310245 | 9.60E-25 | 4.39E-24 |
| NRG3 | 0.320036 | 1.272436 | 1.991289 | 1.12E-29 | 7.10E-29 |
| SPHK1 | 0.608732 | 1.930841 | 1.66535 | 6.24E-19 | 1.81E-18 |
| IL24 | 0.398832 | 0.874173 | 1.132139 | 6.65E-19 | 1.91E-18 |
| RACK1 | 119.6127 | 273.8791 | 1.195169 | 1.90E-35 | 2.92E-34 |
| FAS | 4.339167 | 10.13104 | 1.223293 | 1.32E-26 | 6.75E-26 |
