# Supplementary material for: Establishment and Validation of a Prognostic Risk Model for Autophagy-Related Genes in Clear Cell Renal Cell Carcinoma
Source: Dis Markers. 2020 Nov 10;2020:8841859. doi: 10.1155/2020/8841859 (PMC7676277; doi:10.1155/2020/8841859)
Supplement: Supplementary 3 — Supplementary Table S3 Univariate Cox regression analysis of DEARGs. [file 8841859.f3.docx]

| ID | HR | HR.95L | HR.95H | pvalue |
| --- | --- | --- | --- | --- |
| BIRC5 | 1.7676 | 1.498787 | 2.084625 | 1.31E-11 |
| BID | 3.521392 | 2.440487 | 5.081038 | 1.71E-11 |
| SPHK1 | 1.683524 | 1.420538 | 1.995197 | 1.85E-09 |
| CX3CL1 | 0.637547 | 0.548749 | 0.740713 | 4.05E-09 |
| EIF4EBP1 | 1.490581 | 1.292148 | 1.719487 | 4.34E-08 |
| VMP1 | 1.579137 | 1.332536 | 1.871374 | 1.34E-07 |
| BAG1 | 0.437806 | 0.319963 | 0.59905 | 2.43E-07 |
| CASP4 | 2.392784 | 1.709005 | 3.350144 | 3.75E-07 |
| CDKN2A | 1.562201 | 1.267502 | 1.925418 | 2.88E-05 |
| ERBB2 | 0.649957 | 0.529981 | 0.797093 | 3.50E-05 |
| BNIP3 | 0.706875 | 0.59596 | 0.838433 | 6.79E-05 |

**Univariate Cox regression analysis of DEARGs**
