# Supplementary material for: Establishment and Validation of a Prognostic Risk Model for Autophagy-Related Genes in Clear Cell Renal Cell Carcinoma
Source: Dis Markers. 2020 Nov 10;2020:8841859. doi: 10.1155/2020/8841859 (PMC7676277; doi:10.1155/2020/8841859)
Supplement: Supplementary 4 — Supplementary Table S4 Multivariate Cox regression analysis of 8 genes. [file 8841859.f4.docx]

| id | coef | HR | HR.95L | HR.95H | pvalue |
| --- | --- | --- | --- | --- | --- |
| BID | 0.65442 | 1.924027 | 1.243292 | 2.977483 | 0.00331 |
| CX3CL1 | -0.26844 | 0.764573 | 0.641673 | 0.911012 | 0.002679 |
| EIF4EBP1 | 0.152608 | 1.164868 | 0.991792 | 1.368148 | 0.062951 |
| VMP1 | 0.277954 | 1.320425 | 1.102852 | 1.580921 | 0.002481 |
| SPHK1 | 0.158109 | 1.171294 | 0.955943 | 1.435158 | 0.127187 |

**Multivariate Cox regression analysis of 8 genes**
