# Supplementary material for: Establishment and Validation of a Prognostic Risk Model for Autophagy-Related Genes in Clear Cell Renal Cell Carcinoma
Source: Dis Markers. 2020 Nov 10;2020:8841859. doi: 10.1155/2020/8841859 (PMC7676277; doi:10.1155/2020/8841859)
Supplement: Supplementary 5 — Supplementary Table S5 The correlation between gene signature and clinical traits. [file 8841859.f5.docx]

| id | age | gender | grade | stage | T | M | N |
| --- | --- | --- | --- | --- | --- | --- | --- |
| BID | -0.37  (0.712) | -0.6  (0.549) | -4.025  (7.631e-05) | -5.553  (7.868e-08) | -4.454  (1.39e-05) | -2.87  (0.006) | -5.347  (7.43e-05) |
| CX3CL1 | -1.077  (0.283) | 3.183  (0.002) | 3.563  (4.402e-04) | 3.625  (3.66e-04) | 3.838  (1.761e-04) | 1.284  (0.205) | 1.424  (0.176) |
| EIF4EBP1 | -2.227  (0.027) | 0.761  (0.448) | -3.258  (0.001) | -4.415  (1.519e-05) | -3.693  (2.761e-04) | -3.741  (3.433e-04) | -2.681  (0.017) |
| VMP1 | -1.941  (0.054) | 2.239  (0.026) | -1.448  (0.149) | -2.084  (0.038) | -2.416  (0.017) | -1.908  (0.062) | -0.351  (0.731) |
| SPHK1 | -0.358  (0.721) | -1.25  (0.213) | -4.906  (1.713e-06) | -4.309  (2.587e-05) | -3.928  (1.237e-04) | -3.476  (0.001) | -3.238  (0.006) |
| riskScore | -1.131  (0.260) | 0.686  (0.494) | -4.948  (1.874e-06) | -4.925  (2.489e-06) | -4.483  (1.751e-05) | -2.431  (0.019) | -2.918  (0.011) |

**The clinical correlation of the gene signature**
